# Traffic-Emitted Amines Promote New Particle Formation at Roadsides

**DOI:** 10.1021/acsestair.5c00119

**Published:** 2025-07-16

**Authors:** James Brean, Federica Bortolussi, Alex Rowell, David C. S. Beddows, Kay Weinhold, Peter Mettke, Maik Merkel, Avinash Kumar, Shawon Barua, Siddharth Iyer, Alexandra Karppinen, Hilda Sandström, Patrick Rinke, Alfred Wiedensohler, Mira Pöhlker, Miikka Dal Maso, Matti Rissanen, Zongbo Shi, Roy M. Harrison

**Affiliations:** † Division of Environmental Health and Risk Management, School of Geography, Earth and Environmental Sciences, 1724 University of Birmingham, Birmingham B15 2TT, U.K.; ‡ Department of Chemistry, University of Helsinki, Helsinki 00560, Finland; § Atmospheric Microphysics Department (AMD), 28397 Leibniz Institute for Tropospheric Research (TROP.O.S), Permoserstr. 15, 04318 Leipzig, Germany; ∥ Atmospheric Chemistry Department (ACD), Leibniz Institute for Tropospheric Research (TROP.O.S), Permoserstr. 15, 04318 Leipzig, Germany; ⊥ Aerosol Physics Laboratory, 7840 Tampere University, Tampere 33720, Finland; # Department of Applied Physics, Aalto University, Espoo 11000, Finland; ¶ Physics Department, TUM School of Natural Sciences, Technical University of Munich, Garching 85748, Germany; ∇ Atomistic Modelling Center, Munich Data Science Institute, Technical University of Munich, Garching 85748, Germany; ○ Munich Center for Machine Learning (MCML), Munich 80538, Germany; ⧫ Department of Environmental Sciences, Faculty of Meteorology, Environment and Arid Land Agriculture, King Abdulaziz University, Jeddah 21589, Saudi Arabia

**Keywords:** aerosol, NPF, traffic, pollution, nucleation

## Abstract

New particle formation (NPF) is a major source of atmospheric aerosol particles, significantly influencing particle number concentrations in urban environments. High condensation and coagulation sinks at highly trafficked roadside sites should suppress NPF due to the low survival probability of clusters and new particles, however, observations show that roadside NPF is frequent and intense. Here, we investigate NPF at an urban background and roadside site in Central Europe using simultaneous measurements of sulfuric acid, amines, highly oxygenated organic molecules (HOMs), and particle number size distributions. We demonstrate that sulfuric acid and amines, particularly traffic-derived C_2_-amines, are the primary participants in particle formation. C_2_-amine concentrations at the roadside are enhanced by over a factor of 4 relative to the background, overcoming the effect of enhanced coagulation and condensation sinks. Using machine learning we identify a further but uncertain enhancing role of HOMs. These findings reveal the critical role of traffic emissions in urban NPF.

## Graphical abstract

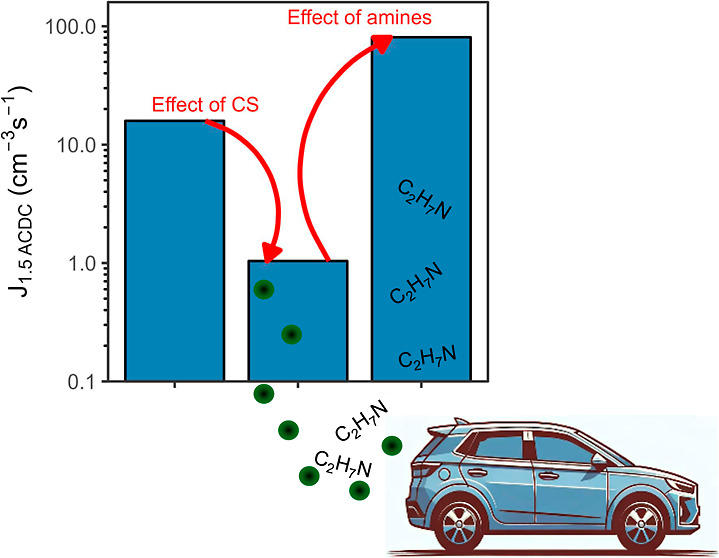

## Introduction

Particulate matter (PM) is the pollutant with the single greatest impact on human health.^1^ The main driver of health outcomes is particle mass; however, it is emerging that particle number concentrations are also important due to the high number count and diffusivity of small particles.^2^ PM also constitutes the single greatest source of uncertainty in radiative forcing in climate models globally^3^ as it can increase radiative forcing by absorbing radiation, decrease it by reflecting radiation, or by acting as cloud condensation nuclei (CCN), affecting cloud albedo and lifetime.

Particle counts in polluted urban air primarily hinge on two processes. First, the emission of particles from processes like traffic and wood burning, and second, new particle formation (NPF). NPF is the generation of particles from gases and is distinct from primary particle emissions. NPF occurs as a burst in particle numbers at 1.5 nm followed by their growth to larger sizes, and these bursts take place on up to 40% of days in certain European cities as an annual average.^4^ Primary particle number emissions peak in traffic rush-hour periods, while NPF typically occurs at late morning to midday.^5^ Chamber studies and urban measurements with chemical ionization mass spectrometer (CIMS) instruments have shown that NPF is initiated by H_2_SO_4_, which forms stable clusters with strong bases such as dimethylamine (DMA), forming new particles at 1.5 nm.^5–8^ Chamber studies indicate a further role of urban highly oxygenated organic molecules (HOMs) in particle formation.^7^ These particles then grow primarily by the condensation of further H_2_SO_4_, alongside HOMs,^9–13^ and other acids and bases,^14^ propelling particle growth to larger sizes (∼20–100 nm).^7^

The rate of generation of new particles is quantified as the particle formation rate (*J*), and is calculated from the particle number size distribution (PNSD). *J* is expected to be suppressed in polluted environments due to high particle surface area concentrations, which are associated with high coagulation sinks and condensation sinks (CSs), efficiently scavenging new particles and precursor gases.^15^ However, long-term analyses of PNSD data sets show that *J* values in polluted environments such as roadsides and megacities are some of the greatest measured worldwide.^4,15^

Traffic emissions of sub-3 nm particles can be broken down into primary particles, which are directly emitted into to the atmosphere from the exhaust, and delayed-primary particles, which are formed in the diluting exhaust, directly following emission.^16^ These are distinct from particles from NPF, which is typically a regional phenomenon. The fraction of sub-3 nm particles which come from primary, delayed primary, and NPF is uncertain, but an important aspect of urban air quality. Recent analyses have shown both that the growth of new particles is more rapid at roadsides compared to surrounding areas because of traffic-related HOMs,^13^ and that traffic is a source of a diverse range of nitrogen-containing species,^17^ but the characteristics of initial particle formation at roadsides has not been investigated in detail.

In this study, we aim to quantify the contribution of traffic emissions to initial particle formation at roadsides. By leveraging simultaneous measurements of sulfuric acid, amines, HOMs, and PNSDs at both a background and roadside site, we assess the relative importance of traffic emissions in shaping early particle formation dynamics.

## Methods

### Measurements

All measurements were taken during a summertime field campaign in Leipzig, Germany, from 2022/08/01 through 2022/08/23. Location of study site shown is shown in Figure S1. The urban background data was collected at an atmospheric research station operated by the Leibniz Institute for Tropospheric Research (TROPOS) within Wissenschaftspark Leipzig (N 51°21′09″, E 12°26′04″ 127 m above mean sea level), hereon referred to as simply “background”. Measurements were taken out of a south-facing window on the fourth floor of a research building at 14 m above ground level and at distances >100 m from highly trafficked roads bordering the site.^18^ The Park perimeter includes transport infrastructure (road, rail and tramways), commercial property (restaurants, hotels, a petrol station etc.), residential property, on-street parking, and greenspace.

Roadside aerosol data was obtained from a permanent observation site located on Eisenbahnstraβe, an important connecting road in the east of the city (N 51°20′44″, E 12°24′23″, 120 m above mean sea level), hereon referred to as simply “roadside”. Measurements were taken from an apartment window at 6 m above ground level on the northern side of the street. The street is ∼20 m in width and is flanked by multistorey period buildings, yielding an aspect ratio of 0.90, and experiences 12,000 vehicles per working day.^18^ The station’s immediate surroundings also include two-lanes of traffic (one in each direction of travel), an integrated tramline, on-street parking, two bicycle lanes (one in each direction of travel), two footpaths, and scant vegetation.

### Particle Number Size Distribution

At the background site a dual mobility particle size spectrometer (D-MPSS) collected the particle number size distribution (PNSD) from 3 to 800 nm. This system is comprised of a drier, an in-house constructed particle sizer with two differential mobility analyzer columns leading to two condensation particle counters (CPC 3025 and CPC 3010). The PNSD from 2.5–42 nm was also collected using a neutral cluster and air ion spectrometer (NAIS, Airel, Estonia), which also measures the PNSD of naturally charged ions from 0.8–42 nm, using conductive rubber tubing for the inlet extending 0.6 m from the building façade. No drying was used here. The D-MPSS data has been collected at this site since 2010, while all other data were collected just for the period of the field campaign.

At the roadside site the PNSD from 10 to 800 nm was collected using a CEN/TS 17434:2020-compliant mobility particle size spectrometer (MPSS). This comprised of a drier, an in-house built particle sizer system, and a CPC 3010. The PNSD from 4.5 to 62 nm was collected using a Nano-MPSS (NanoSMPS, TSI, USA), with no drier attached to the inlet. The PNSD below this point were collected using a 3756 CPC with a lower size cutoff (*D*_50_) of 2.5 nm (TSI, USA), and a particle size magnifier (PSM, Airmodus, Oy) attached to a 3775 CPC (TSI, USA). The particle size magnifier was run in continuous flow mode, such that the whole system has a *D*_50_ of 1.5 nm. The difference in concentration measurements between these instruments was used to measure the 2.5–4.5 and 1.5–2.5 nm fractions. These instruments shared an inlet manifold to maximize flows and minimize losses, which extended 1 m out from the building façade. The MPSS data from 10 to 800 nm has been collected at this site since 2011, while all other data were collected just for the period of the field campaign.

Data inversion and diffusive loss corrections for the D-MPSS at the background and the MPSS at the roadside were done manually. Due to software constraints, the inversion and internal instrument diffusive losses for the TSI Nano-MPSS was done within the AIM10 software separately, while inlet loss corrections were done manually. For both instruments, the total counts for the short column were corrected to that of the long-column by the ratio of counts at 40 nm to harmonize the size distributions. The black carbon (BC) concentration was measured by Multi Angle Absorption Photometer (MAAP).

### Sulfuric Acid, HOMs, and Bases

The University of Birmingham (UoB) and University of Tampere (TAU) CIMS instruments were operated with Eisele-type inlets using nitrate charger ions to measure strong acids and oxygenated organic molecules.^10^ Both instruments were calibrated side-by-side before the campaign using the updated methodology of Mettke et al. (2023).^19^ HOMs were classified into volatility classes following the methodology of Qiao et al (2021).^11^ At the roadside and background sites, 575 and 552 HOMs with ≥5 carbons and ≥4 oxygens were identified, respectively.

The instrument is capable of measuring amines down to ppt levels^20^ clustered with the nitrate dimer and trimer. Correlations between dimer and trimer concentrations are shown in Figure S2, full time series in Figure S3, and peak fits for all three species clustered with both the nitrate dimer and trimer at the background site where amine concentrations were lower in Figure S4. For all peaks, the separation from the closest adjacent peaks is ≥1 half width at full maximum, and the uncertainty due to mass calibration uncertainty for all amines was within 10% of fitted intensity.^21^ We use the lower of the two calibration coefficients from ref 20 for amines to provide a lower limit. A detailed description of the instruments and methodologies is found in ref 13 both CIMS instruments used inlets of ∼1 m which extended 0.7 m from the building façade to minimize boundary-layer effects associated with disturbed flow near surfaces.

### Particle Formation Rates

#### Measured

The formation rate of new particles at size *d*_p_ (*J*_dp_) is calculated as follows.

1 $$J_{d_{p}}=\frac{{dN}_{d_{p}}}{dt}+{CoagS}_{d_{p}}.N_{d_{p}}+\frac{GR}{\Delta d_{p}}.N_{d_{p}}$$

where the first term on the right-hand side comprises the rate at which particles enter the size *d_p_*, and the second term refers to losses from this size by coagulation, *CoagS*_dp_ being the coagulation sink at size *d*_p_, and *N*_dp_ being the number of particles at size *d*_p_, with the third term referring to losses from this size by growth, where the growth rate of new particles can be calculated from the PNSD as follows

2 $$GR=\frac{dd_{p}}{dt}$$

In the instance of this work, the growth rates used to calculate *J* are computed from the modeled rates of ref 13 at both sites we calculated the formation rate at 3 nm using the size bins from 3 to 10 nm, denoted *J*_3_. At the roadside, we calculate the formation rate at 1.5 nm using the size bins from 1.5 to 10 nm, while at the background *J*_3_ was converted to a formation rate at 1.5 nm using the method of ref 22 This is denoted *J*_1.5_.

#### Modeled

We modelled the formation of clusters of sulfuric acid and bases using the Atmospheric Cluster Dynamics Code (ACDC)^23^ running inside the Atmospherically Relevant Chemistry and Aerosol box model (ARCA box).^24^ ACDC models the formation and destruction of molecular clusters using evaporation rate coefficients derived from formation free energies calculated by quantum chemical methods. Here we use the formation free energies calculated with DLPNO–CCSD­(T)/aug-cc-pVTZ//ωB97X-D/6-31++G** level of theory from Myllys et al. (2019).^25^ We used the mean 24 h cycle on NPF days of sulfuric acid, C_2_-amine (here presumed dimethylamine), temperature, relative humidity, and CS to model *J*.

#### Machine Learning

We built a Random Forest (RF) model to simulate the measured *J* values. We also tested Ridge Regressor and Kernel Ridge Regressor models, but the performance was worse for both (Figure S5). We simulated both *J*_3_ and *J*_1.5_. The errors for *J*_1.5_ were greater than for *J*_3_, likely due to uncertainties in quantification (Figure S 6), therefore we utilized *J*_3_. For both the background and roadside site, an individual model was trained. The models predict *J*_3_ based on meteorological variables (solar radiation, pressure, wind speed, temperature, relative humidity, rainfall rate and Haude’s evaporation), NO_2_, NO, BC, the CS, and CIMS measurements. The selected CIMS signals were sulfur-containing species (e.g., H_2_SO_4_, CH_4_SO_3_, SO_5_), iodine-containing species (HIO_3_, INO_3_), amines, formic acid, and HOMs, separated into positive matrix factorization (PMF) factors.

We reproduced the HOM PMF factors reported in Brean et al. (2024)^13^ at 10 min time resolution. These were used in our machine learning (ML) models to represent the HOM signals. Our background ML model includes the factors *spikes*, morning photochemistry 1, midday photochemistry 1, traffic and nighttime. The roadside ML model includes the factors morning photochemistry 1 and 2, midday photochemistry 1 and 2, traffic and nighttime. The diurnal cycle, volatility distribution, and mass defect plots for each factor are shown in Figures S7 and S8.

Data from second to 21st August at 10 min time resolution were used to construct the RF model. To assess the model’s performance with an out-of-sample subset of data, the data sets were randomly split into a training set (80%) and a test set (20%). The total number of observations in both training sets was 2289. The models were trained on the common logarithm of *J*_3_, to reduce the impact of outliers in the model and to stabilize the model variance. The preprocessing details are discussed in the Supporting Information.

We utilized the RandomForestRegressor implementation from scikit-learn.^26^ The hyperparameters of the models are optimized with a 5-fold cross-validation random search implemented by scikit-learn (RandomizedSearchCV), given its efficiency in tuning multiple hyperparameters.^27^ The selected hyperparameters were the number of trees, the depth of each tree, the minimum number of samples per leaf, the minimum number of samples required at each internal node, and the maximum number of variables per tree (tuned hyperparameters in Table S2). To evaluate model performance, we used the mean absolute error (MAE).

*J*_3_, the target variable, is log-transformed. For the model features, missing values are filled with the nearest nonmissing value. The total number of missing values is small. A stationary transformation is then applied, which detrends, deseasonalises the signal, and applies a Box–Cox transformation if the signal is heteroscedastic. This transformation has a greater impact on roadside signals than background signals, suggesting that nonstationary information is more critical at roadside sites. To prevent information loss, the stationary transformation is applied only to the background model. Finally, outliers are detected using an isolation forest algorithm, and the values are standardized.

We quantify the effect of measured HOMs, meteorological data and other chemical species by conducting a feature importance investigation. To this end, the models are interpreted using SHapley Additive exPlanations (SHAP).^28^ SHAP locally interprets ML models by assessing each input variable’s contribution to the target prediction (in this study, *J*_3_). In this study, we employ the Python-based TreeExplainer,^29^ a SHAP implementation specifically designed for tree-based models such as RF.

## Results

### Roadside Sulfuric Acid and Amines

The mean diel cycle in the PNSD at both the background and roadside sites shows traffic peaks at diameters between 5 and 30 nm (∼06:00 and 18:00), as well as NPF beginning at 09:00 and growing throughout the day (Figure 1a). Concentrations of particles at all sizes are greatest at the roadside site. Diel sulfuric acid concentration peaks at 1 × 10^6^ cm^–3^ and 2 × 10^6^ cm^–3^ at the background and roadside site respectively (Figures 1b and S10). Nighttime sulfuric acid is roughly a factor of 2 higher at the roadside compared to the background, and the ratio of sulfuric acid dimer/monomer is greater by a factor of 2 at the roadside (23:00 to 03:00 concentration 3 × 10^4^ cm^–3^ and 5 × 10^4^ cm^–3^ at the background and roadside site respectively, Figure 1b). We could resolve the sulfuric acid trimer during NPF periods at the background site, but not at the roadside (Figure S9).

**Figure 1:**
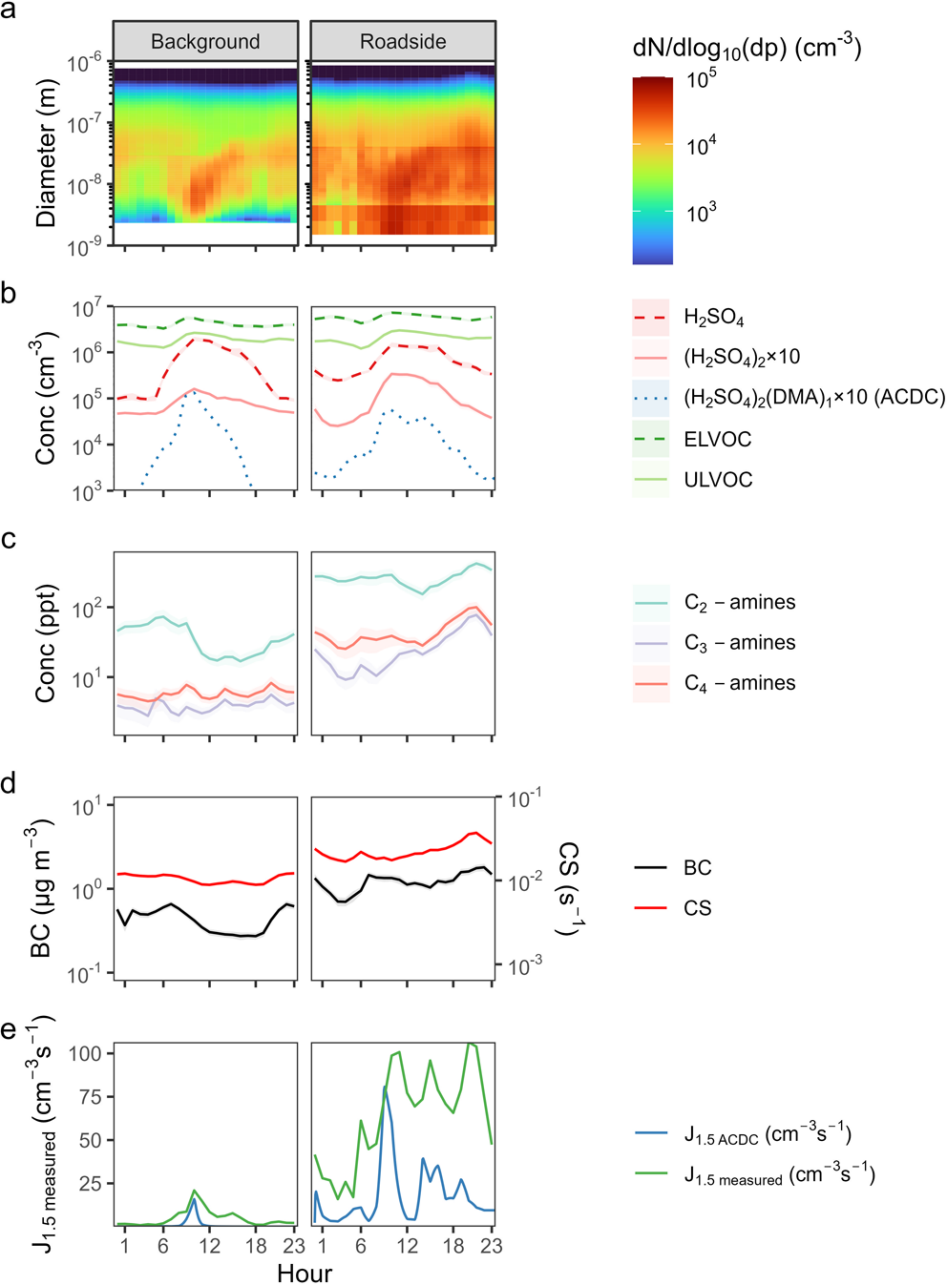
Diel cycles of particles and gases at the background and roadside sites: (a) the particle number size distribution; (b) concentrations of sulfuric acid dimer and monomer, as well as extremely low volatility organic compounds (ELVOC) and ultralow volatility organic compounds (ULVOC) as measured by NO_3_^–^ CIMS, alongside the ACDC-modeled sulfuric acid dimer-DMA cluster concentration; (c) amines as measured by NO_3_^–^ CIMS; (d) black carbon, measured by MAAP and condensation sink (CS) as calculated from the particle number size distribution; (e) modeled and measured J_1.5_ from ACDC and the particle number size distribution, respectively.

HOMs are binned into volatility classes, of which, the concentrations of extremely low volatility organic compounds (ELVOC) and ultralow volatility organic compounds (ULVOC) are greater at the roadside by factors of 2 and 1.5, respectively (Figures 1b and S10). The concentration of C_2_, C_3_, and C_4_ amines, here presumed to be dimethylamine (C_2_H_7_N), trimethylamine (C_3_H_9_N), and diethylamine (C_4_H_11_N) in the gas phase are greater at the roadside than the urban background by a factor of 4.6, with the concentration of C_2_ amines being the greatest (Figures 1c and S10). C_2_ amines are thought to come primarily from road traffic emissions,^30,31^ and they peak concurrently with black carbon (BC) concentrations and CS (*R* ^2^ = 0.17 between amines and BC, Figure S11), which are also greatest at the roadside (factors of 3.6 and 1.7, respectively, Figures 1d and S10).

The diel cycle of *J*_1.5_ based on measurement and modeling (with ACDC) is presented in Figure 1e. *J*_1.5_ was modeled using the mean diel cycle of sulfuric acid, C_2_-amine (assumed to be dimethylamine), temperature, relative humidity, and CS on NPF days. The hourly mean peak in daytime measured *J*_1.5_ at the roadside and background are 107 and 21 cm^–3^ s^–1^, respectively. The hourly mean peak in ACDC-modeled *J*_1.5_ is within 25% of the measured *J*_1.5_ at both the background and the roadside, indicating that sulfuric acid and amine nucleation dominates *J*_1.5_ at midday.

While the ACDC-modeled *J*_1.5_ increases slightly during the morning at both sites, it lacks the sharp rush-hour peaks seen in observations. The peak in BC occurs around 07:00, but the modeled *J*_1.5_ peaks two to 3 h later, at 09:00 at the roadside and 10:00 at the background site. At the time of peak BC, the modeled *J*_1.5_ values are 80-fold (roadside) and 24-fold (background) lower than their respective maxima. This discrepancy likely reflects the absence of primary particles and some combustion-derived vapors in the model input. Although elevated morning amine concentrations may enhance early cluster formation in the model, as shown by the elevation to (H_2_SO_4_)_2_·DMA and *J*_1.5_ at 06:00 at the roadside, the dominant factor driving ACDC results appears to be photochemical H_2_SO_4_ production. In contrast, the observed *J*_1.5_ peaks are more consistent with contributions from direct or delayed primary particle emissions. We also cannot discard any contributions of delayed primary or primary particle emissions to *J*_1.5_ during NPF, especially at the roadside.

### Formation Rates of Particles

The sulfuric acid dimer concentration shows a positive correlation with the C_2_-amine concentration as measured by the NO_3_^–^ CIMS (Figure 2a). The dimer-to-monomer ratio is elevated at the roadside relative to the background, consistent with higher C_2_-amine concentrations promoting cluster stability. However, this ratio is lower than that observed in the CLOUD chamber at 278 K and at lower CS values^32^ (Figure 2b).

**Figure 2:**
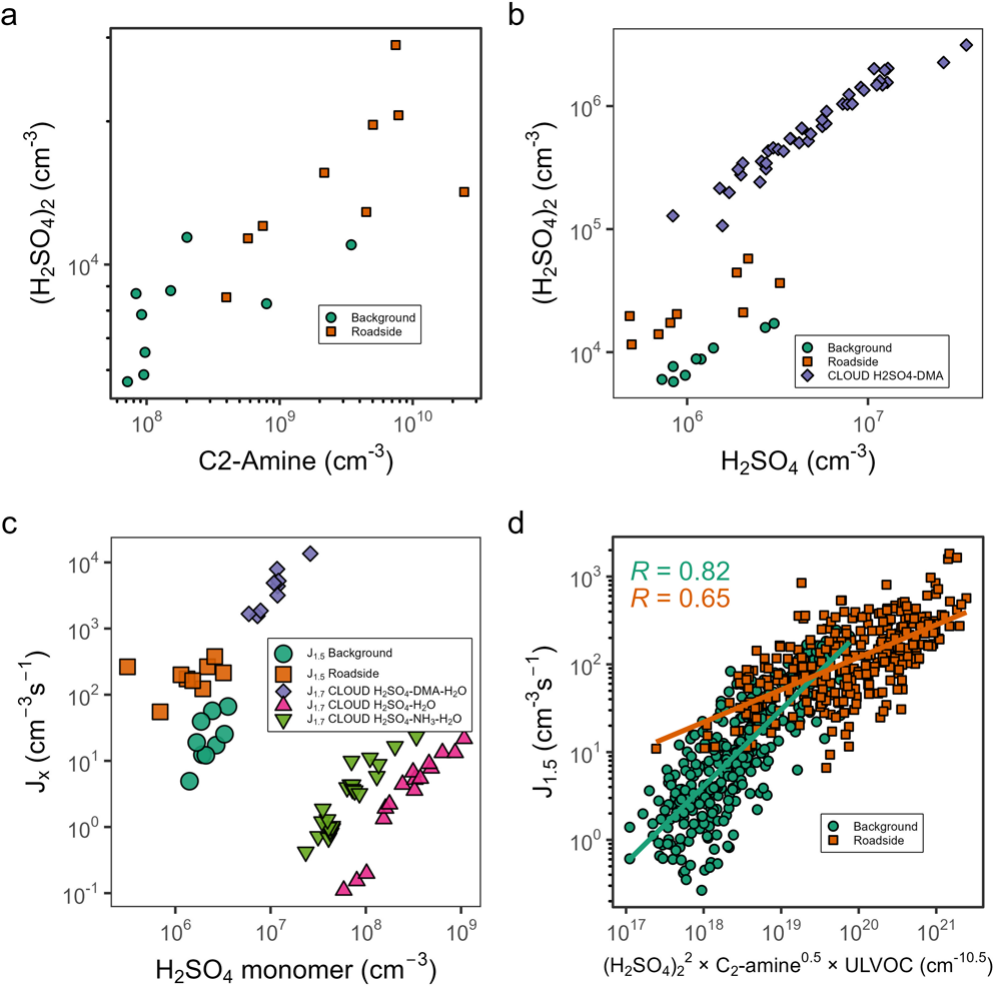
Mechanism of particle formation at the roadside and background: (a) relationship between H_2_SO_4_ dimer and C_2_-amines as measured by NO_3_^–^ CIMS; (b) H_2_SO_4_ dimer plotted against H_2_SO_4_ monomer from our measurements, alongside CLOUD chamber data from ref 32 (c) *J*_1.5_ and *J*_1.7_ versus H_2_SO_4_ from our measurements, as well as CLOUD chamber studies. Purple points from ref 36 pink and light green points from ref 33 (d) *J*_1.5_ plotted versus (H_2_SO_4_)_2_^2^·C_2_-amine^0.5^·ULVOC for both measurement sites.

Relative to sulfuric acid concentrations, our measured *J*_1.5_ values, are greater than the *J*_1.7_ values observed in the CLOUD chamber with sulfuric acid alone, as well as sulfuric acid in the presence of NH_3_.^33^ However, our *J*_1.5_ values align with the *J*_1.7_ values observed in the CLOUD chamber under conditions of sulfuric acid and high concentrations of dimethylamine (DMA) (Figure 2c),^34^ despite elevated temperatures. At both measurement sites, the best fit for *J*_1.5_ was achieved using a modified expression from Lehtipalo et al.:^35^ (H_2_SO_4_)_2_^2^·C_2_-amine^0.5^·ULVOC (Figure 2d). Excluding either the amines or the ULVOCs resulted in a poorer fit. The reduced fit accuracy at the roadside, compared to the background, is likely attributable to the greater influence of the production of delayed-primary particles from traffic, elevating *J*_1.5_.^16^

### Drivers of Formation Rate Variability

We utilized a RF model to identify the role of HOMs, acids, bases, pollutant emissions, and meteorology in the formation of new particles. For the RF models at both the roadside and background site, the MAE decreases with increasing training size (Figure 3a). There is no noticeable difference between the learning rate of the models at the two sites, and the variance remains constant with increasing training size. The roadside data are noisier with frequent spikes due to local traffic, hindering a more accurate prediction. The model performs consistently across all training set sizes as the variance is not high for any given training size.

**Figure 3:**
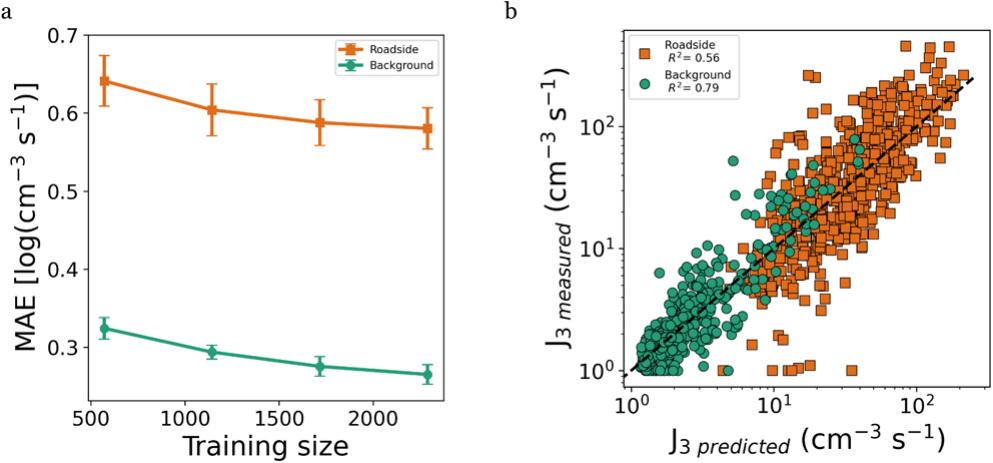
Quantitative performance of the machine learning models. (a) Learning curve for the mean absolute error (MAE) of the *J*_3_. For each training size the mean value and variance are obtained by training the model five times by randomly reshuffling the data set; (b) correlation between the measured and predicted J_3_ (cm^–3^ s^–1^) of the best performing models of the five data set random reshuffles for the background site and roadside site.

While there is a slight overprediction of low *J*_3_ values, and slight underprediction for high values, the background model achieves a good overall performance (*R* ^2^ = 0.79, Figure 3b). By contrast, the roadside model performs worse (*R* ^2^ = 0.56, Figure 3b). The values lower than ∼30 cm^–3^ s^–1^ are overpredicted by the RF model. For higher values, the predicted values show a greater spread than for the background site.

SHAP is a model interpretability method that quantifies the contribution of each input variable to the RF model’s prediction at a given time point. It assigns each feature a value called a SHAP value that represents how much that feature increased or decreased the predicted *J*_3_ relative to the model’s average prediction. This allows us to identify not just which variables are important overall, but how they influence the model’s output under different atmospheric conditions. At the background site three sulfur-containing species—H_2_SO_4_, (H_2_SO_4_)_2_, and SO_5_—dominate *J*_3_ prediction (Figure 4a), alongside solar radiation, which is highly correlated with the sulfur containing species. High concentrations of these species are generally associated with higher predicted *J*_3_, although SO_5_ may be a fragment or reactive intermediate.^37^ Lower measured C_2_H_7_N is associated with elevated *J*_3_, although C_2_H_7_N has been shown to decrease during NPF events, likely as it is efficiently incorporated into nucleating clusters.^38^ Elevated atmospheric pressure, associated with stable meteorology and high solar radiation, is associated with higher predicted *J*_3_, while elevated RH is associated with lower *J*_3_. Elevated HOMs from the PMF factor from *midday photochemistry factor* is mostly associated with elevated *J*_3_. NO_2_ is also associated with elevated *J*_3_. Less impactful are INO_3_, temperature, and the PMF factors *morning photochemistry 1*, *nighttime chemistry*, and *spikes*.

**Figure 4:**
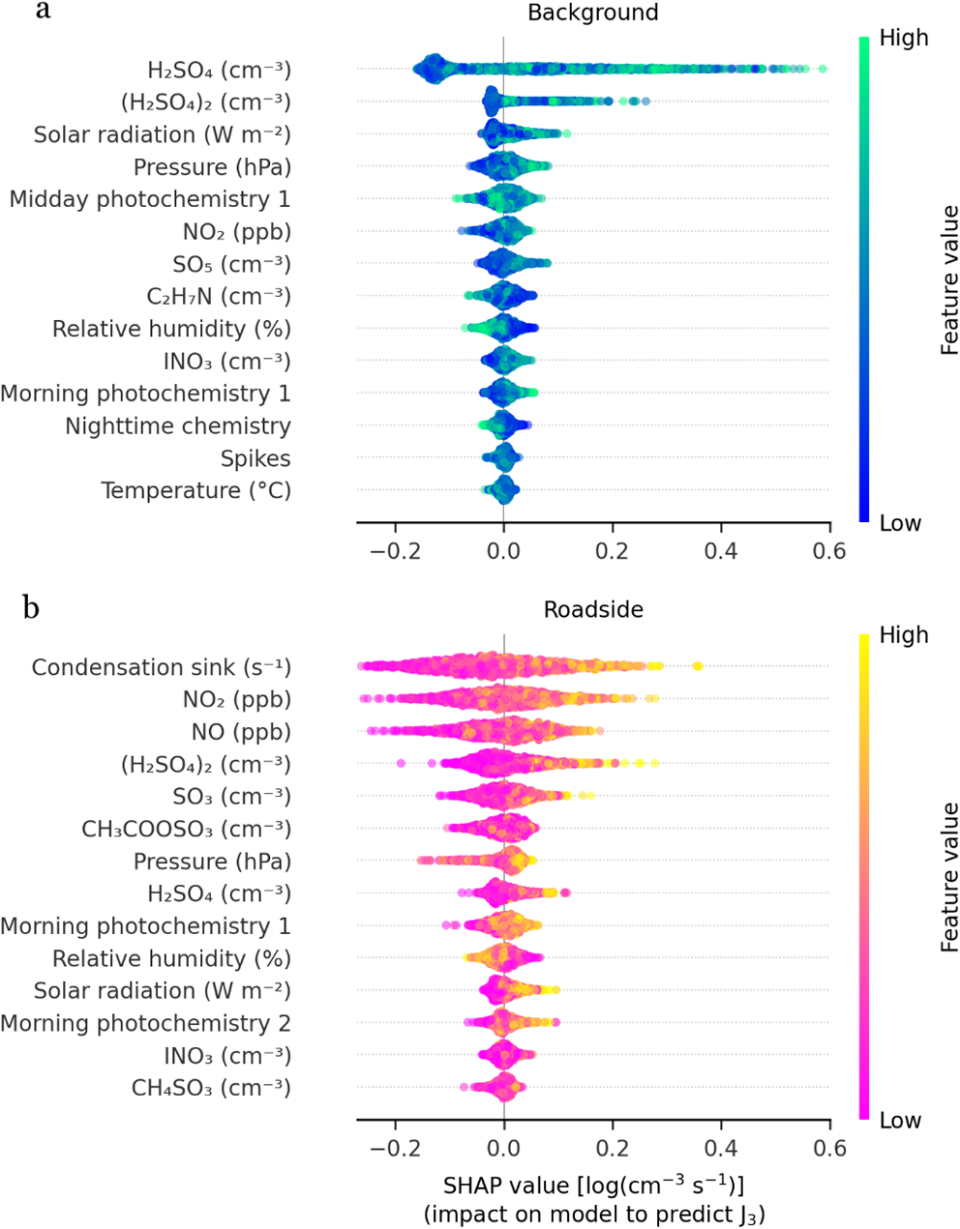
Top 14 most impactful features for the prediction of *J*_3_ with the RF models. (a) SHapley Additive exPlanations (SHAP) values for the background model; (b) SHAP values of the roadside model. Higher SHAP values indicate that the feature increased the model’s prediction of *J*_3_, with color showing whether high or low values of the feature caused that effect. The features are ranked by their highest mean |SHAP| values, with only the top 14 shown. Midday photochemistry, morning photochemistry, and nighttime chemistry refer to the HOM PMF factors of ref 13 A SHAP value of 0 represents the average *J*_3_ value. Positive SHAP values indicate an increase in *J*_3_, while negative values indicate a decrease. The spread of SHAP values and the color gradient show the strength and direction of the correlation, with a clear gradient suggesting a strong positive or negative relationship.

At the roadside site (Figure 4b), CS, NO, and NO_2_ are the primary predictors of *J*_3_, with higher CS and NO_*x*_ associated with higher *J*_3_. Similarly, NO and NO_2_ are important for the *J*_3_ prediction, collectively indicating that primary traffic emissions substantially contribute to *J*_3_ at the roadside, even during NPF event periods. (H_2_SO_4_)_2,_ H_2_SO_4_, SO_5_, and the *morning photochemistry 1* PMF factor are all positively related with *J*_3_, while CH_3_COOSO_3_ has no clear gradient. Consistent with the background site, elevated RH is inversely related with *J*_3,_ and solar radiation shows a slight positive correlation.

## Discussion

### Amines and Roadside NPF

Long-term measurements and intensive field campaigns show that NPF occurs more intensely at roadsides than at urban background sites.^4,39–42^ We show that at a moderately trafficked roadside site in urban Europe, amines, CS, and HOMs are all enhanced relative to a nearby urban background site (Figures 1 and S10). Our ACDC modeling results show that while the enhanced CS suppresses *J* values, the enhancement to amine concentrations enhances *J* values, resulting in a balancing effect. This is visualized in Figure 5. If we reduce the concentration of C_2_-amines in ACDC to that of the background while maintaining the higher CS, formation rates decrease to 1 cm^–3^ s^–1^, lower than typically observed in any urban environment,^5^ and akin to that seen in remote environments where vapor concentrations are low,^38^ however, also incorporating the roadside concentration of C_2_-amines then enhances the nucleation by 2 orders of magnitude. This demonstrates that the elevated amine concentrations are necessary to overcome the elevated CS for NPF to proceed at roadsides.

**Figure 5:**
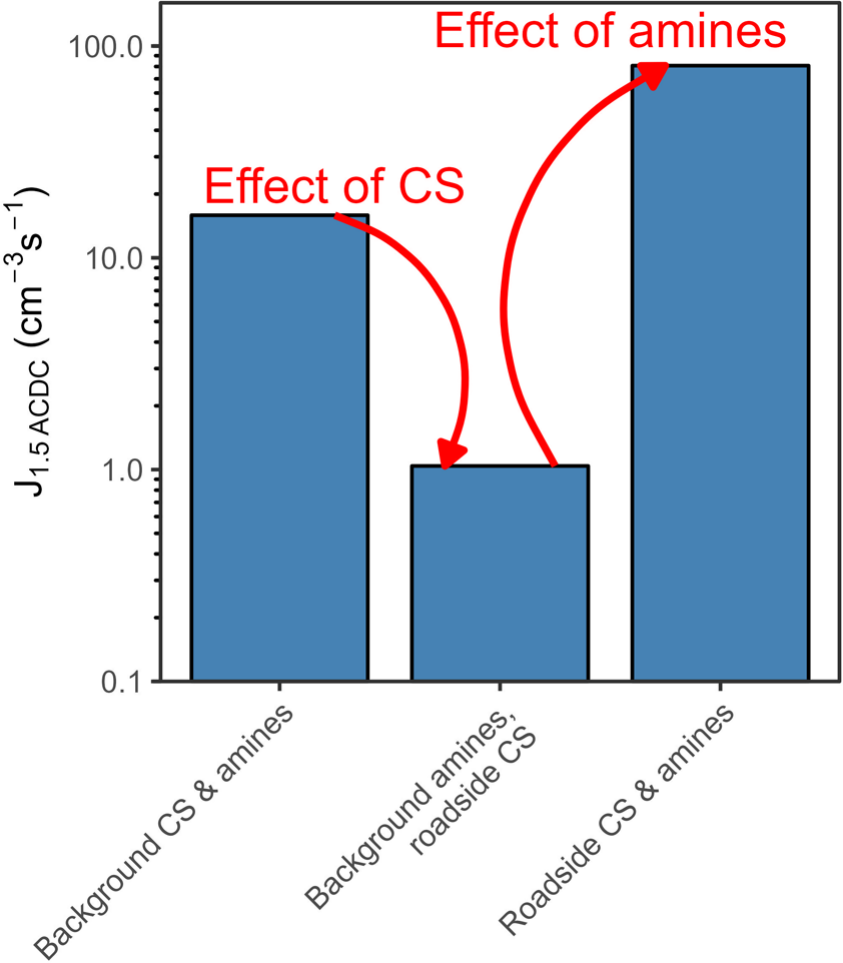
Simulated particle formation rates under varying scenarios. Hourly daytime maxima in *J*_1.5_ are presented. *J*_1.5_ was modeled using the mean cycle of sulfuric acid, C_2_-amine (assumed to be dimethylamine), temperature, relative humidity, and condensation sink on NPF days at each site. The leftmost bar represents background conditions, the rightmost bar depicts roadside conditions, and the middle bar shows roadside conditions adjusted to have the same amine concentrations as the background.

Our peaks in C_2_ amines are concurrent with BC emissions (Figure 1d). It is likely that our measured C_2_-amines are primarily emitted, and are likely dimethylamine rather than ethylamine.^30^ This is corroborated by source-apportionment work in Beijing, showing that traffic is the primary source of C_2_-amines,^31^ and measurement in Houston, showing that amine concentrations correlate with CO concentrations.^43^ Trimethylamine has been shown to accelerate NPF in urban environments,^44^ and comes from septic system emissions alongside traffic, but our observed C_3_-amine concentrations are low. Similarly, diethylamine enhances nucleation of sulfuric acid to a potentially greater degree than dimethylamine,^45^ and gasoline engines are a known diethylamine source, but our observed diethylamine concentrations are low, and therefore they play a much more minor role in NPF than dimethylamine. Dimethylamine is therefore the most likely base to stabilize our observed sulfuric acid clusters.

We demonstrate that NPF is primarily driven by sulfuric acid and amines based on several lines of evidence. First, sulfuric acid dimer concentrations and the dimer/monomer ratio are elevated at the roadside (Figure 1b), scaling with C_2_-amine levels (Figure 2a), which are more than four times greater than at the background site (Figure 1c). This ratio, a proxy for cluster stability, is lower than in Almeida et al. (2013)^32^ but reflects the higher temperature and CS in our data. Second, a sulfuric acid trimer peak appears only during NPF events (Figure S9), further indicating the presence of strongly bound sulfuric acid-amine clusters at the background site. The NO_3_^–^ CIMS fragments larger clusters, and this fragmentation is sensitive to instrument tuning, which could explain why this peak was not resolved at the roadside, and why larger sulfuric acid–amine clusters were not observed. Third, our nucleation rates align with chamber data from Kürten et al. (2018),^36^ even at warmer temperatures (Figure 2c), with the effect of elevated temperature possibly being compensated for by the enhancing effect of ULVOC. Fourth, a linear fit of *J*_1.5_ versus (H_2_SO_4_)_2_^2^·C_2_-amine^0.5^·ULVOC (Figure 2d) supports both the role of sulfuric acid and amines, and the role of ULVOCs in stabilizing clusters, as their inclusion improves the fit. Fifth, sulfuric acid and amines are key predictors of *J*_3_ in the RF model (Figure 4). Finally, simulated nucleation rates of sulfuric acid and dimethylamine from ACDC closely match the observed 09:00 *J*_1.5_ peak at both sites, though the model underestimates traffic-related peaks at the roadside (Figure 1e). Collectively, these findings confirm that nucleation proceeds via sulfuric acid and amines, with HOMs, specifically ULVOCs likely contributing to cluster stability.

We achieve a good match between our observed *J*_1.5_ values and the *J*_1.7_ values of Kürten et al. (2018)^36^ (Figure 2c). Our observations were conducted at higher temperatures than those in the CLOUD chamber which would typically reduce *J*_1.5_ values. However, the presence of other contributing species such as C_3_ and C_4_-amines,^44,45^ as well as HOMs,^7,46^ and small concentration of iodine oxoacids^47^ (peak diel HIO_3_ 1.4 × 10^5^ cm^–3^ and 8 × 10^4^ cm^–3^ at background and roadside, respectively), likely enhances *J*_1.5_, resulting in the observed agreement.

While the quantification of amines in the NO_3_^–^ CIMS is uncertain and we did not directly calibrate for DMA in the CIMS, the two CIMS instruments were voltage-tuned to operate similarly,^13^ with similar ratios of charger ion clusters in the instrument (e.g (HNO_3_)_2_NO_3_^–^:HNO_3_NO_3_^–^). We use the same calibration coefficient for amines for both instruments, even though the roadside instrument was more sensitive to sulfuric acid.^13^ Our reported ratio of roadside/background amine concentrations is therefore a lower limit.

### Understanding the Drivers of New Particle Formation Rates

Chamber observations indicate that high HOM concentrations can increase nucleation rates of sulfuric acid and amines,^7^ although this is likely highly dependent on the present functional groups.^48^ Similarly, HOMs, specifically ULVOCs can condense down onto freshly nucleated particles, enhancing *J*_3_.^9,49^ Due to the uncertainty in the structures and functionalities of observed HOMs, alongside their high number, there are no robust parametrizations of SA-Amine-HOM nucleation that can be replicated in ACDC.^50^ Moreover, the interactions between J_3_ and HOM concentrations are also likely nonlinear. We therefore can only provide inferential evidence about the roles of HOMs in our observed nucleation processes.

To elucidate this uncertain role of HOMs, they were separated into the PMF factors of Brean et al. (2024),^13^ which describe different sources of HOMs dictated by oxidant concentration, termination reaction, and/or VOC source. These were then incorporated them into our RF model. At the background site, the *midday photochemistry 1* factor was the most important for the prediction of *J*_3_, while at the roadside (Figure 4), the *morning photochemistry 1* factor was most important (Figure S7). The latter of these has a large contribution from HOMs with high carbon numbers (>20) in the ULVOC range (Figure S8), further implying the role of ULVOCs in particle formation. The ELVOC and ULVOC signals in our data show less diel variation than sulfuric acid, which may explain their relatively smaller SHAP values (Figures 1b and 4). As the contribution of HOMs to *J*_3_ is highly dependent on the structure of the HOM, future work should focus on the individual HOM signals.

SHAP values indicate that C_2_-amines are inversely related with *J*_3_ in the background RF model, and this may be because gas-phase C_2_-amines are incorporated into new clusters during periods of high *J*_3_.^38^ The RF model also highlights acetic sulfuric anhydride (measured as CH_3_COOSO_3_^–^) in our data—likely the reaction product of acetic acid and SO_3_ as a key predictor of *J*_3._ Recent laboratory studies show that SO_3_ can react with a range of acids in the atmosphere to form sulfuric anhydrides,^51,52^ while field studies have measured high concentrations of SO_3_ and its reaction products in urban environments,^51,53^ indicating that this reaction channel may be important for NPF in polluted environments.

The ML models can predict *J*_3_ with a fair performance. The difference in performance between the background model and the roadside model (Figure 3) are likely as the features describing primary emissions (BC, CS, NO, NO_2_) do not adequately describe the sub-10 nm particle emissions from traffic. For instance, CS may not fall within the size range relevant for *J*_3_, NO_2_ is generally a secondary indicator, and BC mass often reflects aged primary emissions. Further, the ratio of sub-10 nm particle emissions to these proxies may vary differently with vehicle type, fuel type, and dilution. This would be mitigated with higher time resolution measurements, as well as a wider suite of emission proxies. Time-series data inherently contain complex and nonlinear relationships with *J*_3_. Even with the limited data set currently available, ML reveals valuable clues; however, much longer data coverage would likely further enhance model performance and our understanding of the dynamics governing primary and secondary new particle formation.

### Diurnal Cycles in Particle Formation Rates

At the roadside site, the prediction of *J*_3_ is highly dependent on the CS, NO, and NO_2_, with high values associated with high *J*_3_. The diurnal cycle of measured *J*_1.5_ exhibits peaks both at in the early morning, evening, and at 8:00–09:00; the latter peak is consistent with the observed start-time of NPF in Leipzig (Figure 1a),^13,54^ and also the predicted peak in sulfuric acid-amine nucleation via ACDC (Figure 1e). Conversely, the early morning and evening-time peaks are likely dominated by primary (or delayed-primary) traffic emissions. This is corroborated by the ML model, in which much of the observed variance in roadside *J*_3_ can be modeled with CS, NO, and NO_2_ alone (Figure 4b).

However, we cannot exclude the contribution of directly emitted sub-3 nm particles from traffic, even during peak NPF hours. These primary or delayed-primary particles may be emitted concurrently with gaseous precursors such as amines and sulfuric acid, potentially acting additively to enhance apparent NPF. Our ACDC simulations, while capturing the timing of *J*_1.5_, still underestimate its magnitude, suggesting a role for primary particles not included in the model, and this is most prevalent at the roadside site.

As our measurement site is representative of a typical roadside environment, our results therefore confirm speculation that the typical “triple-humped” diurnal cycle observed in PNSD measurements at roadsides is dominated by traffic in the morning and evening, and NPF at late morning to midday.^4,39,40,55–57^ Specifically, traffic-related primary emissions dominate in the morning and evening, while NPF takes over from late morning to midday, although it is highly likely that our measured late morning NPF contains a substantial contribution of primary and delayed-primary particles.

## Atmospheric Implications

These findings have significant implications for future air quality and climate scenarios in urban regions. As traffic patterns evolve through increased urbanization, shifts to electric vehicles, and changes in emission regulations, the contribution of NPF to particle numbers may shift. For instance, reductions in amine emissions from advanced vehicle technologies or stricter controls on traffic-related volatile organic compounds, precursors to HOMs, could diminish roadside NPF rates, potentially lowering particle number concentrations. Conversely, in growing megacities with persistent or increasing traffic density, where NPF is already a major source of particle mass,^58–60^ NPF could become an even larger source of ultrafine particles, exacerbating air quality challenges. Given that small particles are highly diffusive and potentially more harmful to human health than larger PM per unit mass,^2^ and that they influence climate through radiative forcing and cloud condensation nuclei (CCN) activity,^5^ understanding and predicting these trends, especially the fractions of particles from primary versus secondary sources, is important for improving air quality management, assessing health risks, and refining climate models.

## Supplementary Material



## Notes

Eleven Figures and 3 Tables. Experimental site map; time series and correlation plots of amines; amine peak fitting and clustering data; hyperparameter ranges and tuning results for machine learning models; learning curves for model performance; PMF factor diurnal cycles, mass defect and volatility plots; sulfuric acid trimer peak fits; concentrations of NPF-relevant species on NPF days; black carbon–amine emission correlations (PDF)
