# Supplementary material for: Traffic-Emitted Amines Promote New Particle Formation at Roadsides
Source: ACS EST Air. 2025 Jul 16;2(8):1704–13. doi: 10.1021/acsestair.5c00119 (PMC12340761; doi:10.1021/acsestair.5c00119)
Supplement: Supplementary file 1 [file ea5c00119_si_001.pdf]

Supplementary Information for: Traffic-emitted amines promote new particle formation at roadsides

James Brean[1], Federica Bortolussi[2], Alex Rowell[1], David C.S. Beddows[1], Kay Weinhold[3], Peter Mettke[4] Maik Merkel[3], Avinash Kumar[5], Shawon Barua[5], Siddharth Iyer[5], Alexandra Karppinen[5], Hilda Sandström[6], Patrick Rinke[6,7,8,9], Alfred Wiedensohler[3], Mira Pöhlker[3], Miikka Dal Maso[5], Matti Rissanen[2,5], Zongbo Shi[1], Roy M. Harrison[1,10]*

[1] Division of Environmental Health and Risk Management, School of Geography, Earth and Environmental Sciences, University of Birmingham, Birmingham B15 2TT, United Kingdom

[2] Department of Chemistry, University of Helsinki, Helsinki, 00560, Finland

[3] Leibniz Institute for Tropospheric Research (TROPOS), Atmospheric Microphysics Department (AMD), Permoserstr. 15, 04318 Leipzig, Germany

[4] Leibniz Institute for Tropospheric Research (TROPOS), Atmospheric Chemistry Department (ACD), Permoserstr. 15, 04318 Leipzig, Germany

[5] Aerosol Physics laboratory, Tampere University, Tampere, 33720, Finland

[6] Department of Applied Physics, Aalto University, Espoo, 11000, Finland

[7] Physics Department, TUM School of Natural Sciences, Technical University of Munich, Garching, 85748, Germany

[8] Atomistic Modelling Center, Munich Data Science Institute, Technical University of Munich, Garching, 85748, Germany

[9] Munich Center for Machine Learning (MCML), Munich, Germany

[10] Department of Environmental Sciences, Faculty of Meteorology, Environment and Arid Land Agriculture, King Abdulaziz University, Jeddah, 21589, Saudi Arabia

*To whom correspondence should be addressed. **Email:** r.m.harrison@bham.ac.uk

Table S1: Hyperparameter tuning range list for each machine learning model

**Model**	**Hyperparameter**	**Tuning range**	**Info**
Ridge regression	Kernel	linear	Linear kernel
Kernel ridge regression	Kernel	rbf	Gaussian kernel
	Lambda	np.logspace(-10, -1, 10)	Regularization strength
	Sigma	np.logspace(-10, 0, 10)	Length scale
Random forest regressor	Max depth	(4, 30, 2), None	The length of each tree, from the root to the leaves
	Min samples leaf	(2, 10, 1)	Minimum number of samples per leaf
	Min samples split	(2, 10, 1)	Minimum number of samples per split
	N estimators	(50, 301, 10)	Maximum number of estimators
	Max features	(1,14,1)	Size of the subsets of features when splitting a node

Table S2: Hyperparameters tuned for the random forest regressor at the highest training size (2289 observations) for the roadside and background sites.

**Site**	**Random seed**	**Hyperparameters**				
		Max depth	Min samples leaf	Min samples split	N estimators	Max features
Background	42	20	3	8	100	13
	5	22	7	7	260	8
	52	22	4	2	250	12
	1066	20	2	4	90	8
	324	28	2	7	280	7
Roadside	42	14	2	6	180	7
	5	28	4	5	120	13
	52	18	3	5	300	6
	1066	22	2	2	160	13
	324	28	2	3	70	13

Table S3: Hyperparameters tuned for the kernel ridge regressor at the highest training size (2289 observations) for the roadside and background sites.

**Site**	**Random seed**	**Hyperparameters**	
		Lambda	Sigma
Background	42	0.01	0.07742
	5	1e-06	0.07742
	52	1e-10	2.8e-06
	1066	1e-06	0.07742
	324	1e-07	0.07742
Roadside	42	0.001	0.00046
	5	0.01	0.00046
	52	1e-08	2.15e-07
	1066	0.001	0.00046
	324	0.1	0.00046

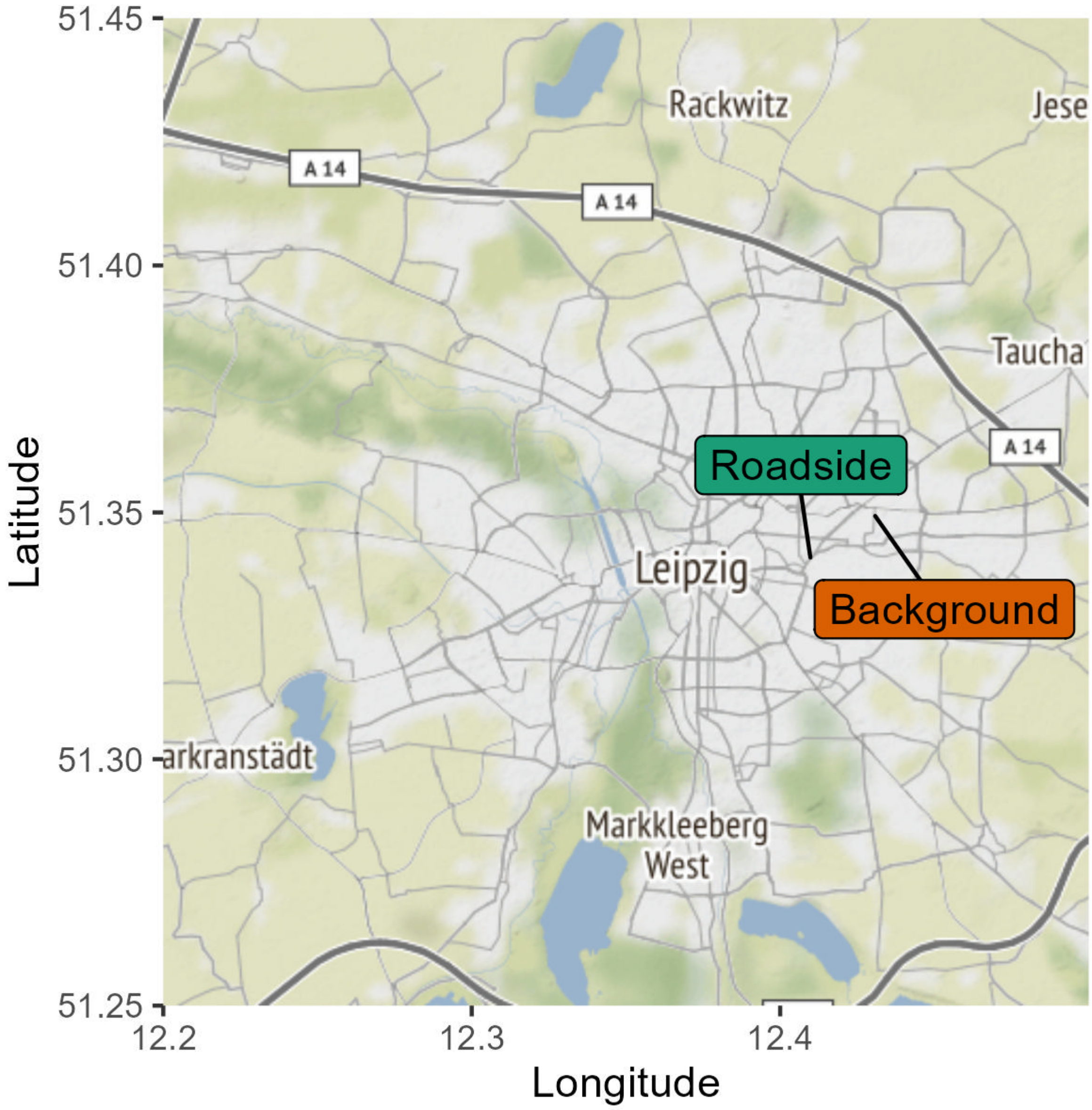

Figure S1: Location of the measurement sites, showing a map of Leipzig, with the roadside and background sites labelled. Map data from Stamen Maps. Reproduced from reference 1. Copyright 2024 American Chemical Society.

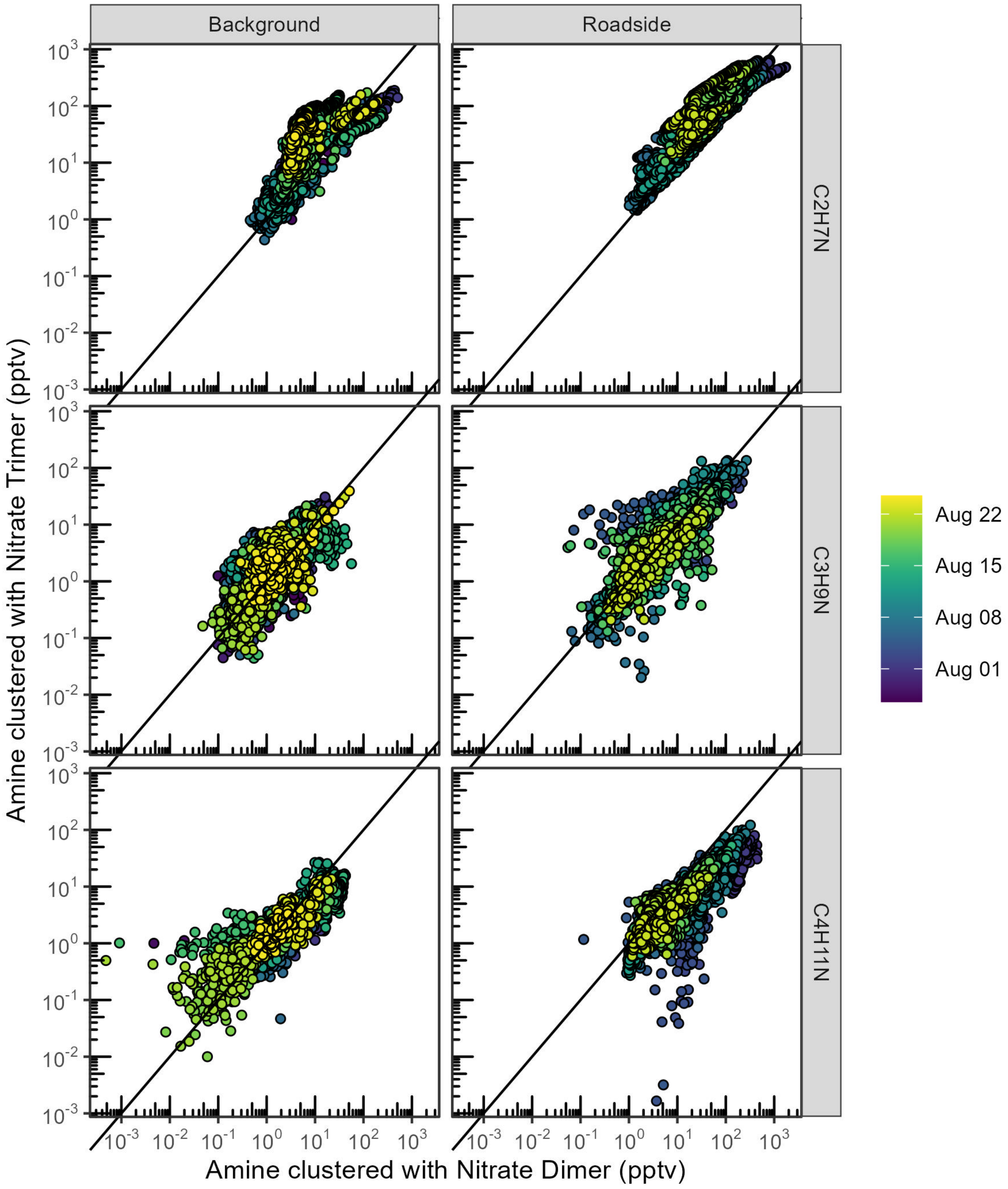

Figure S2: Correlations between C_2, C_3, and C_4 amines in the NO_3^- CIMS as clustered with the nitrate dimer and trimer at each site

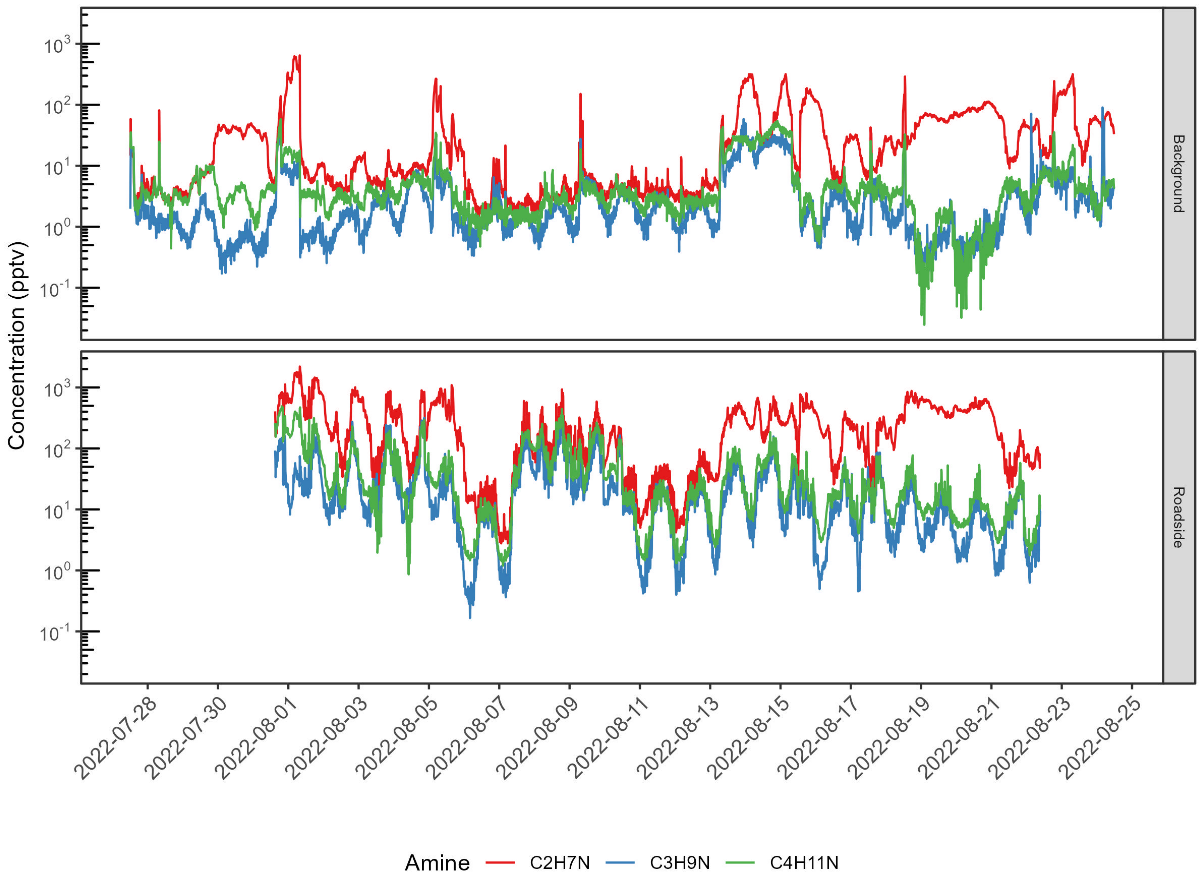

Figure S3: Time series of C_2, C_3, and C_4 amines in the NO_3^- CIMS at each site.

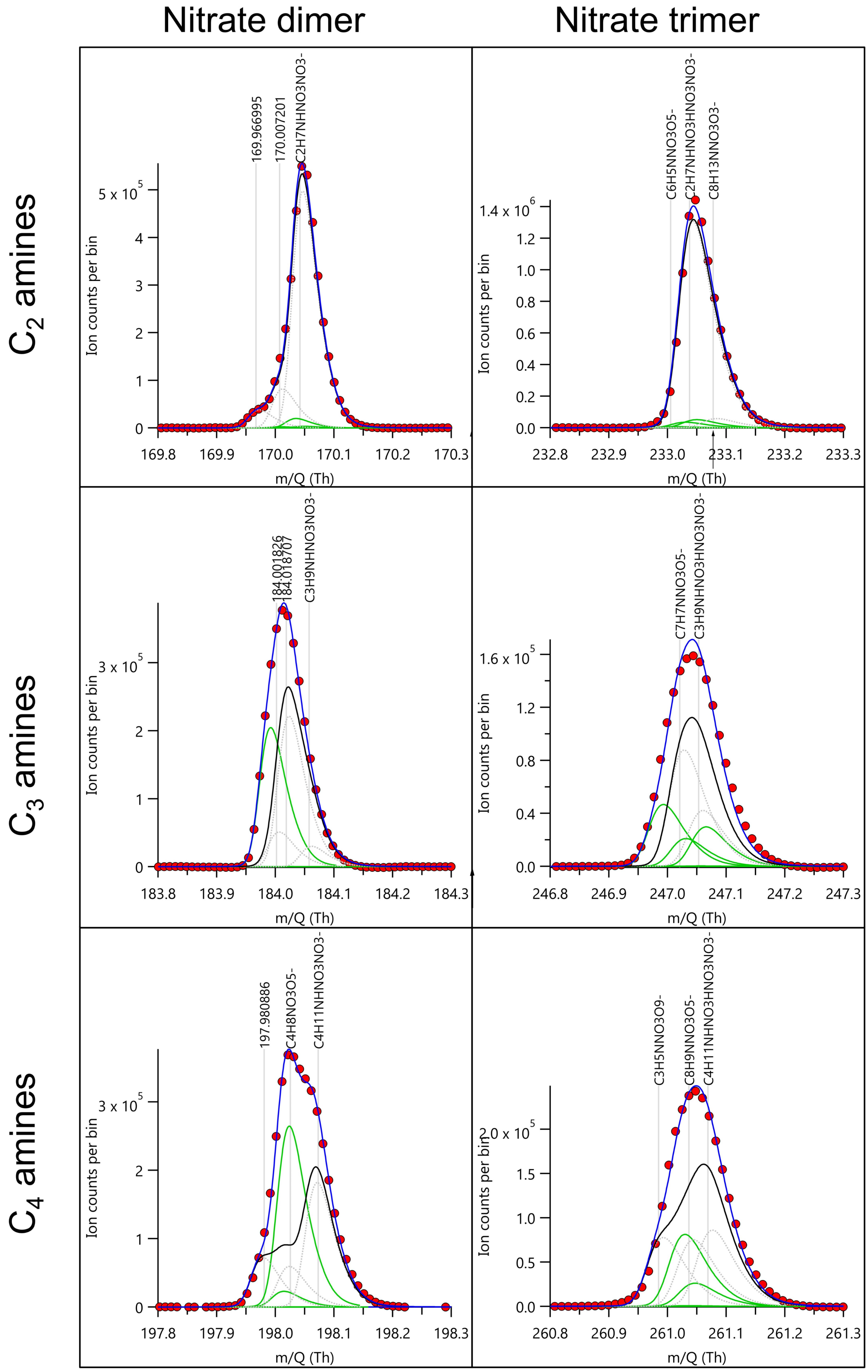

Figure S4: Peak fittings for C_2, C_3, and C_4 amines clustered both with the nitrate dimer and trimer. Peak fits are for the average of whole campaign at roadside site. The red points are raw data, grey lines are the fitted peaks, green lines are isotopes, grey is the sum of fitted peaks, blue line is the sum of fitted peaks and isotopes.

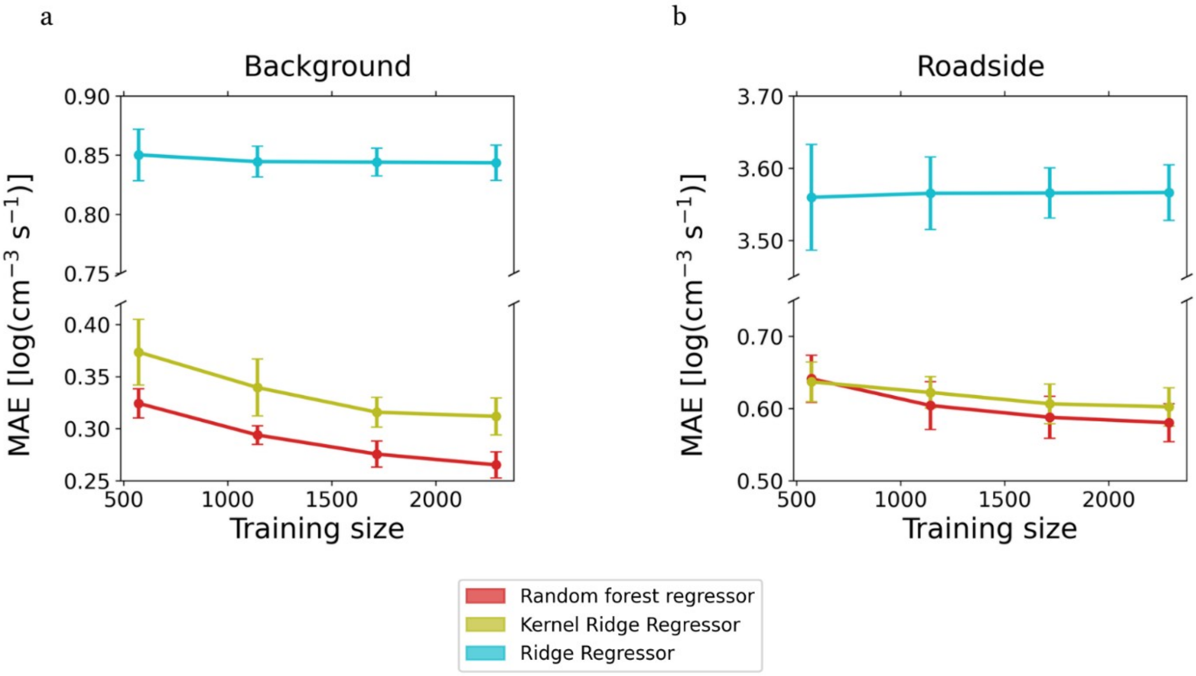

Figure S5: Learning curve with mean absolute error (MAE) of the logarithmic J_3 for three trained models. The x axis reports the training size, and the y axis reports the MAE of J_3 [log(cm^{-3}s^{-1})]. For each training size the mean value and variance are obtained by training the model five times by randomly re-shuffling the dataset.

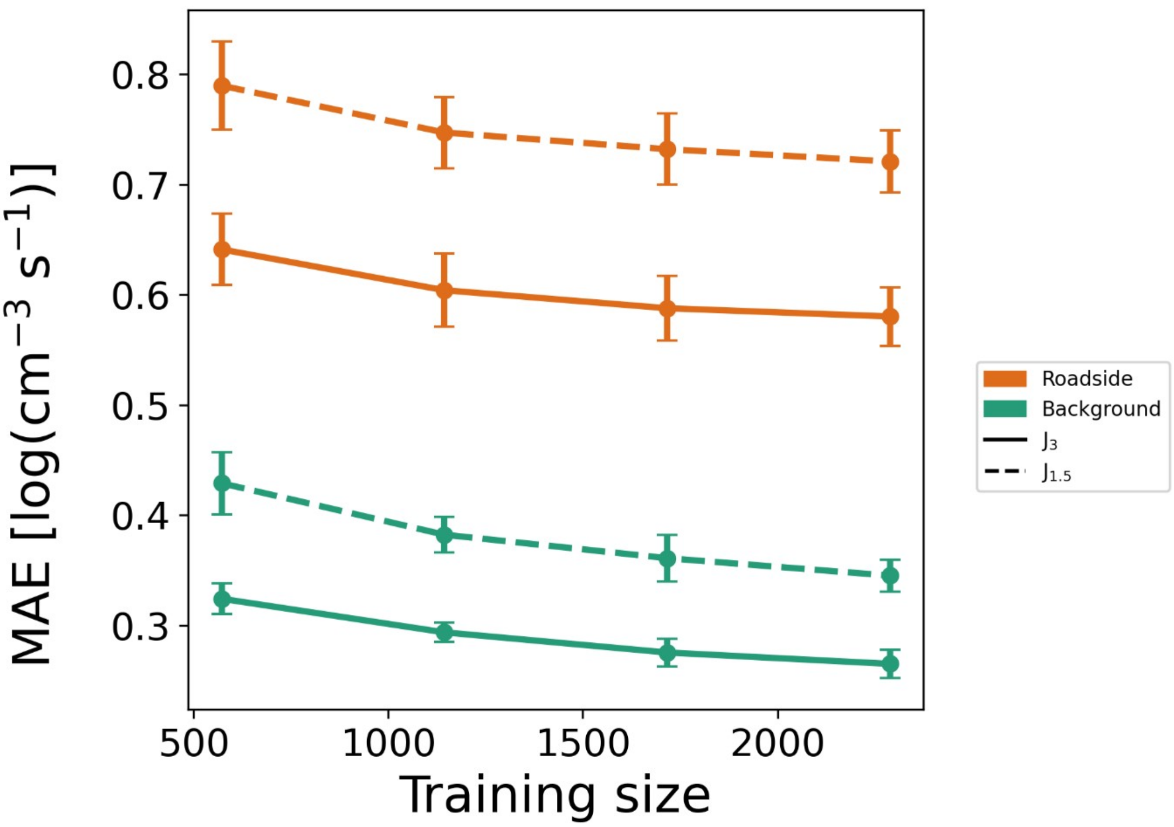

Figure S 6: Learning curve of the random forest models showing the variance of mean absolute error (MAE) of the logarithmic J_3 and $J_{1.5}$ with training size for the roadside and background site. The x axis reports the training size, and the y axis reports the MAE of J_3 and $J_{1.5}$ [log(cm^{-3}s^{-1})]. For each training size the mean value and variance are obtained by training the model five times by randomly re-shuffling the dataset.

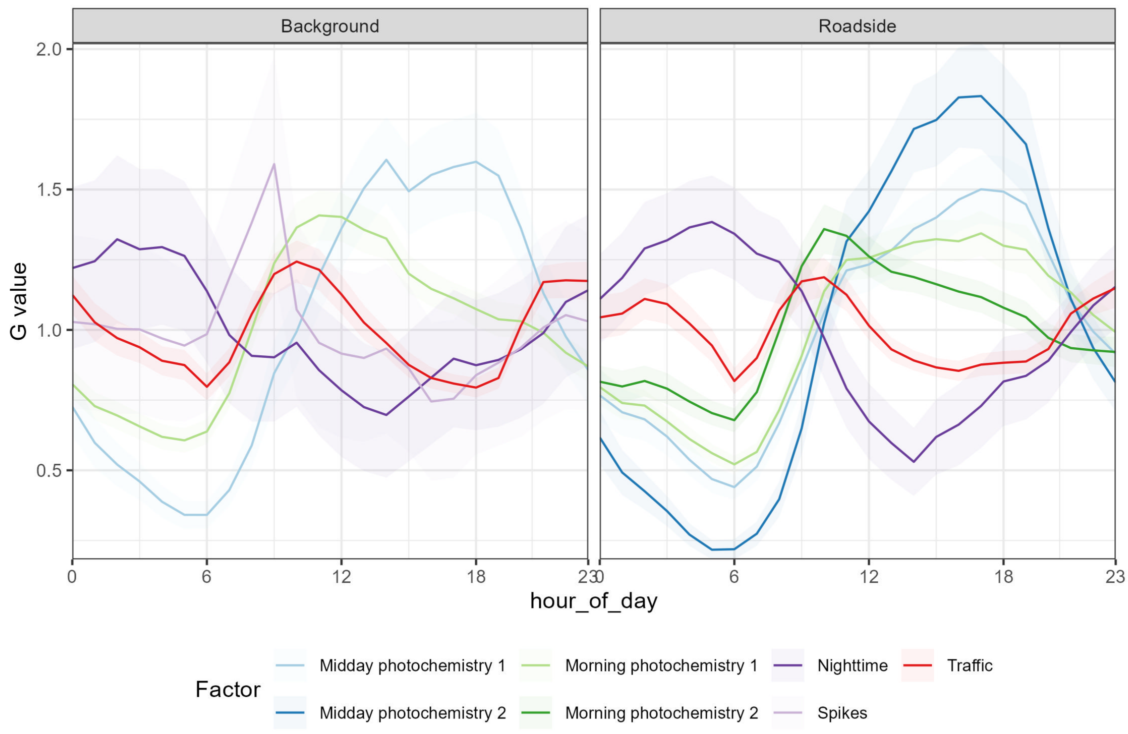

Figure S7: Diurnal cycle of PMF factors

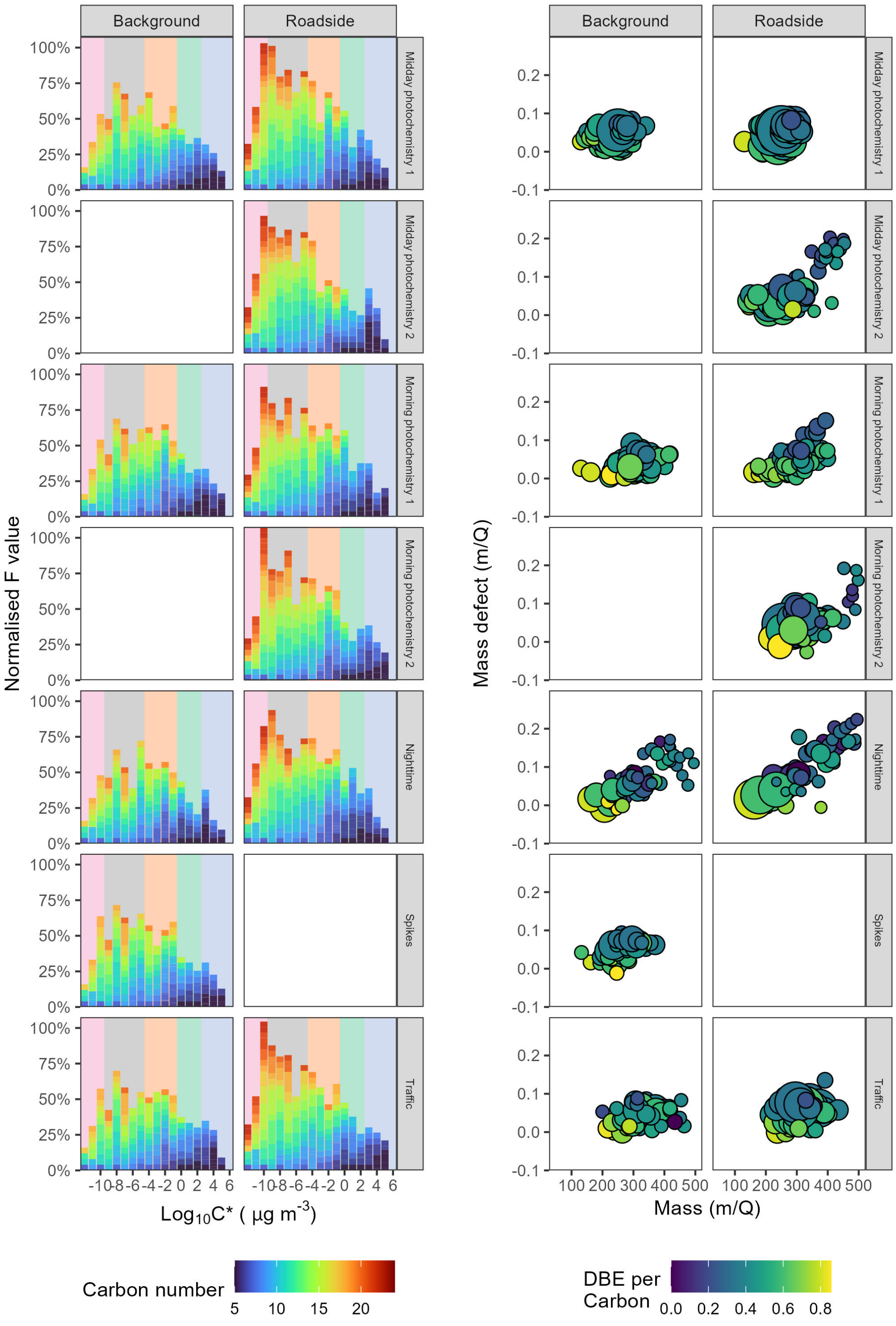

Figure S8: (a) mass defect plots for PMF factors; (b) volatility distributions for PMF factors, akin to plots from Brean et al. (2024) [2]

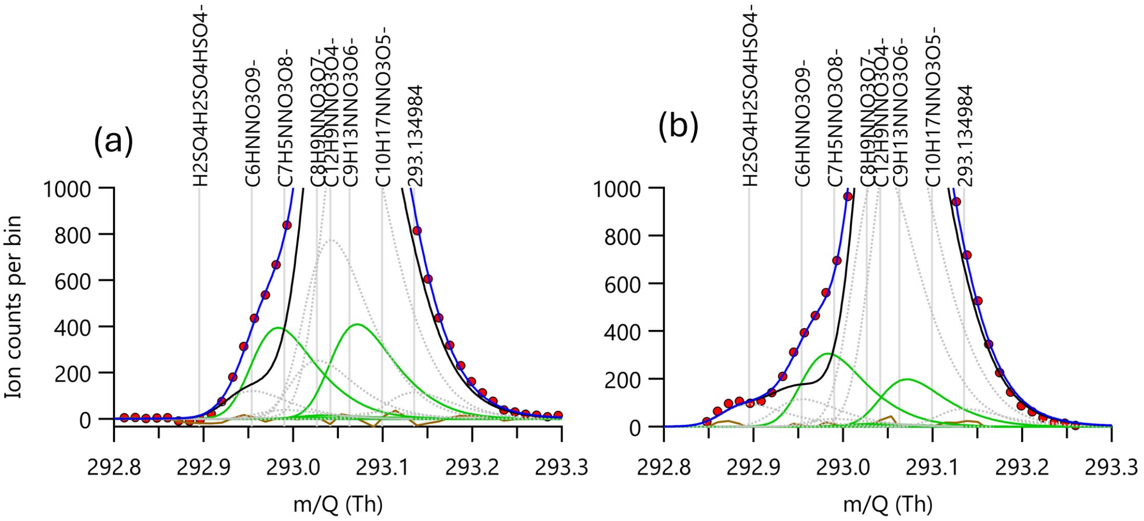

Figure S9: Peak fits for sulfuric acid trimer at nighttime with zero signal (a) and (b) during NPF with signal. Peak fits taken for the 6th August 2021. Before NPF is 00:00 til 04:00, NPF is 08:00 til 13:00 during NPF. Data from Background site. The red points are raw data, grey lines are the fitted peaks, green lines are isotopes, grey is the sum of fitted peaks, blue line is the sum of fitted peaks and isotopes.

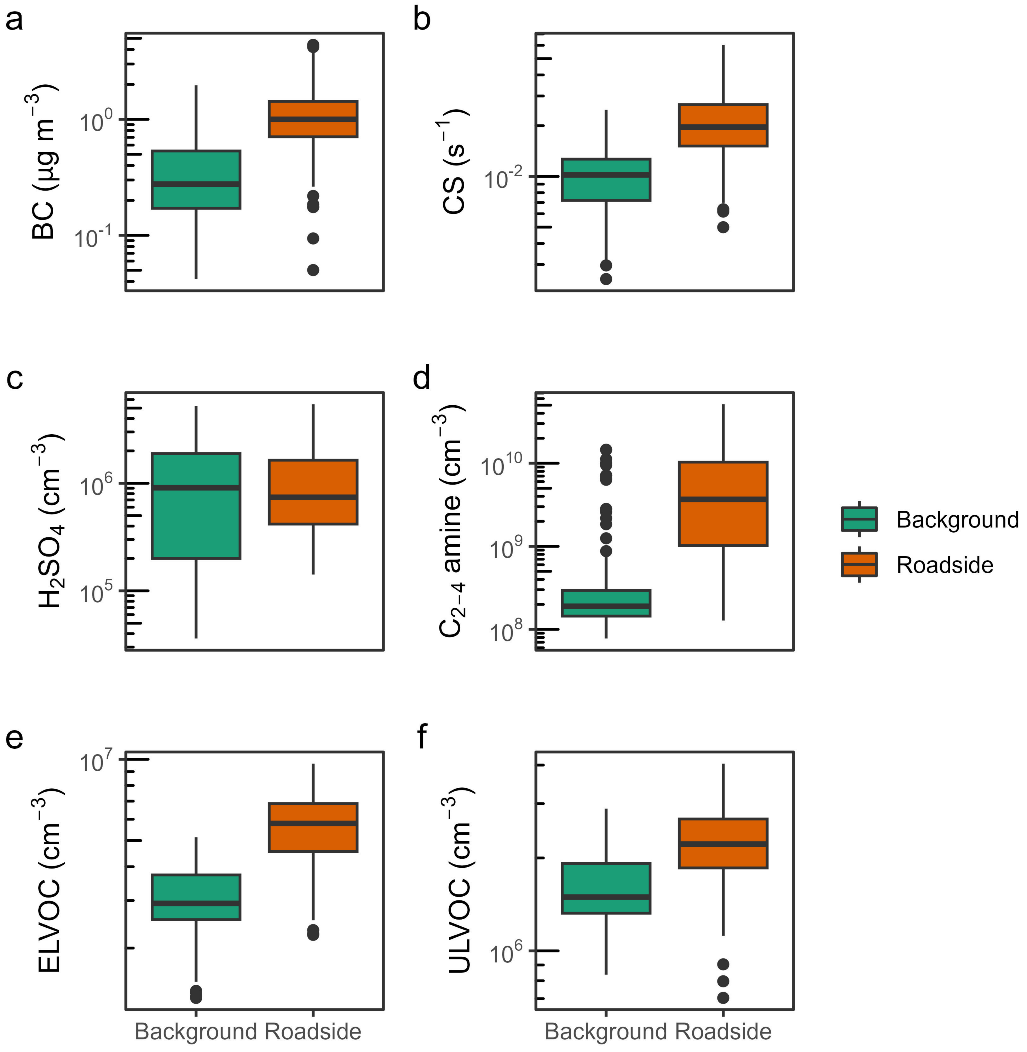

Figure S10: Concentrations on NPF days of (a) black carbon, (b) condensation sink; (c) H_2SO_4, (d) amines, (e) ELVOC, and (f) ULVOC at both the background (green) and roadside (orange) sites. H_2SO_4 is taken between 06:00 and 20:00.

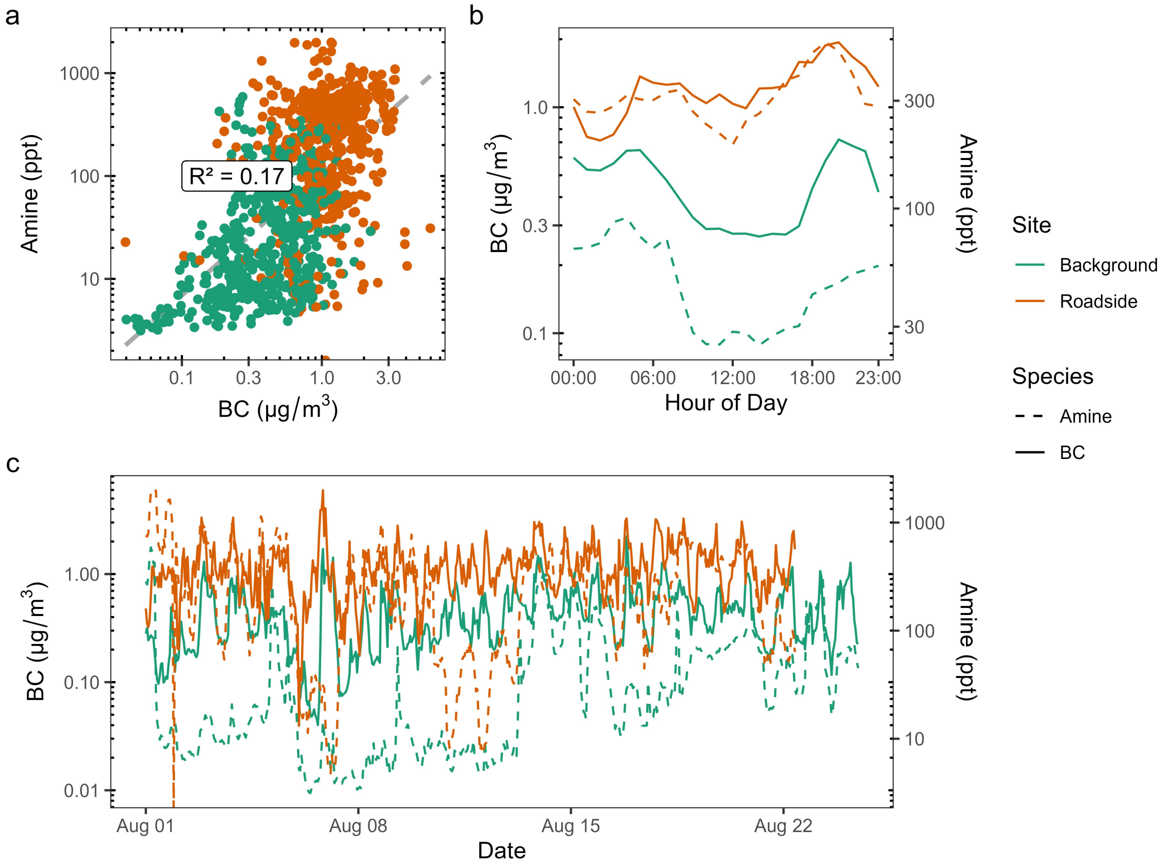

Figure S11: Concurrent emissions of BC and amines, showing (a) the correlation between the sum of C_{2-4} amines and BC, (b) the diurnal cycles of BC and amines across the whole campaign, and (c) the time series of BC and amines.

References

1 Brean, J. *et al.* Road Traffic Emissions Lead to Much Enhanced New Particle Formation through Increased Growth Rates. *Environmental Science & Technology* **58**, 10664-10674, doi:10.1021/acs.est.3c10526 (2024).
